# Thapsigargin at Non-Cytotoxic Levels Induces a Potent Host Antiviral Response that Blocks Influenza A Virus Replication

**DOI:** 10.3390/v12101093

**Published:** 2020-09-27

**Authors:** Leah V. Goulding, Jiayun Yang, Zhimin Jiang, Hongyu Zhang, Daniel Lea, Richard D. Emes, Tania Dottorini, Juan Pu, Jinhua Liu, Kin-Chow Chang

**Affiliations:** 1 School of Veterinary Medicine and Science, University of Nottingham, Sutton Bonington Campus, Sutton Bonington LE12 5RD, UK; leah.goulding@pirbright.ac.uk (L.V.G.); svxjy@exmail.nottingham.ac.uk (J.Y.); richard.emes@nottingham.ac.uk (R.D.E.); tania.dottorini@nottingham.ac.uk (T.D.); 2 Key Laboratory of Animal Epidemiology, Ministry of Agriculture, College of Veterinary Medicine, China Agricultural University, No. 2 Yuanmingyuan West Road, Beijing 100193, China; jiang_zm@cau.edu.cn (Z.J.); zhang_hongyu95@163.com (H.Z.); 08024h@cau.edu.cn (J.P.); ljh@cau.edu.cn (J.L.); 3 Advanced Data Analysis Centre, University of Nottingham, Sutton Bonington Campus, Sutton Bonington LE12 5RD, UK; daniel.lea@nottingham.ac.uk

**Keywords:** thapsigargin, endoplasmic reticulum stress, unfolded protein response, antiviral, influenza A virus, innate immunity

## Abstract

Influenza A virus is a major global pathogen of humans, and there is an unmet need for effective antivirals. Current antivirals against influenza A virus directly target the virus and are vulnerable to mutational resistance. Harnessing an effective host antiviral response is an attractive alternative. We show that brief exposure to low, non-toxic doses of thapsigargin (TG), an inhibitor of the sarcoplasmic/endoplasmic reticulum (ER) Ca^2+^ ATPase pump, promptly elicits an extended antiviral state that dramatically blocks influenza A virus production. Crucially, oral administration of TG protected mice against lethal virus infection and reduced virus titres in the lungs of treated mice. TG-induced ER stress unfolded protein response appears as a key driver responsible for activating a spectrum of host antiviral defences that include an enhanced type I/III interferon response. Our findings suggest that TG is potentially a viable host-centric antiviral for the treatment of influenza A virus infection without the inherent problem of drug resistance.

## 1. Introduction

There is no entirely satisfactory antiviral treatment for influenza A infections. By directly targeting the highly mutable virus, current and near-market antivirals are inherently susceptible to virus resistance [1,2]. Thus, there is an unmet need for antivirals to be clinically effective and less vulnerable to viral mutation. An alternative antiviral approach is to target host factors that facilitate virus replication or promote host immune response [3,4]. Two distinct advantages underpin host-dependent therapies: they (i) do not directly target the virus proteins, thereby reducing selection pressure and the likelihood of drug resistant escape mutants and (ii) can target host factors shared by different viruses [5,6,7]. Therefore, harnessing an effective host antiviral response is potentially a better alternative or even a replacement for existing antivirals.

Thapsigargin (TG) is an inhibitor of the sarcoplasmic/endoplasmic reticulum calcium (Ca^2+^)-ATPase (SERCA) pump; it impedes the replenishment of Ca^2+^ in the endoplasmic reticulum (ER) store resulting in net loss of Ca^2+^ from the ER [8]. ER Ca^2+^ efflux triggers ER stress [9], which involves the unfolded protein response (UPR) that serves to restore ER function and promote cellular survival but under extreme and prolonged stress it can also induce apoptosis. The UPR is orchestrated by three ER resident mediators, activating transcription factor 6 (ATF6), PKR-like ER kinase (PERK), and inositol-requiring enzyme 1 (IRE1) [10,11]. The ER stress response has been found to facilitate virus replication. Influenza A virus replication was reported to be impeded by inhibiting the IRE1 pathway [12]. Foot-and-mouth disease (FMD) virus activates PERK to suppress antiviral type I and III interferon (IFN) response [13]. However, ER stress-activated IRE1α also promotes antiviral RIG-I-IFN-β signalling [14,15,16,17]. Thus, the role of TG mediated ER stress on host innate immunity affecting influenza A virus replication is unclear. Here, we report on the discovery that low non-cytotoxic doses of TG elicit an extended antiviral state against influenza virus replication in respiratory epithelial cells, including primary normal human bronchial epithelial (NHBE) cells, and that its oral administration protects mice against an otherwise lethal virus challenge. We show that TG-induced ER stress leading to UPR is a key innate immune driver that mediates a range host-centric antiviral processes to block virus replication.

## 2. Materials and Methods

### 2.1. Cells and Influenza A Viruses

Primary NHBE cells from three different donors and bronchial epithelial growth media were supplied by Promocell. Immortalised neonatal porcine tracheal epithelial (NPTr) cells, MDCK cells, and Vero cells were cultured in DMEM-Glutamax supplemented with 10% foetal calf serum (FCS) and 100 U/mL penicillin-streptomycin (P/S). Human USSR H1N1 (A/USSR/77), A/Puerto Rico/8/1934 H1N1 (PR8), A/equine/Newmarket/5/03 (H3N8) and A/canine/New York/51864/2008 (H3N8) viruses were used in this study. All viruses were propagated in 10-day-old embryonated chicken eggs, and allantoic fluid was harvested at 72 h post-inoculation. Virus was aliquoted and stored in −80 °C until further use.

### 2.2. Cell Viability and Caspase 3/7 Assays

Cell viability based on the detection of ATP was determined using a CellTiter-Glo Luminescent Cell Viability Assay kit (Promega, Madison, Wisconsin, USA), and activated caspase 3 and 7 were quantified using a Caspase-Glo 3/7 Assay kit (Promega), according to manufacturer’s instructions. Continuous monitoring of cell viability was performed using a RealTime-Glo™ MT Cell Viability Assay (Promega) kit (Promega) according to the manufacturer’s instructions.

### 2.3. Chemical Priming of Cells

TG and tunicamycin (Sigma-Aldrich, St. Louis, MO, USA) were dissolved in DMSO according to manufacturer’s recommendations. The selected concentrations for their antiviral use were chosen for no detectable adverse effect on cell viability. For pre-infection chemical priming, cells were cultured in the presence of the indicated chemical, diluted in the appropriate cell culture media for 30 min, unless otherwise indicated, rinsed three times with PBS, and followed by influenza virus infection as described.

### 2.4. Infection and Progeny Virus Quantification

Infection medium of NPTr cells was Ultraculture medium (Lonza, Basel, Switzerland) supplemented with 100 U/mL P/S, 2 mM glutamine and 250ng/mL l-1-tosylamide-2-phenylethyl chloromethyl ketone (TPCK) trypsin (Sigma-Aldrich). Infection medium of MDCK and Vero cells was OptiMEM (Thermo Fisher Scientific, Waltham, Massachusetts, USA) supplemented with 100 U/mL P/S and 200 ng/mL TPCK trypsin. Infection medium of NHBE cells was bronchial epithelial growth medium (Promocell, Heidelberg, Germany) supplemented with 125 ng/mL TPCK trypsin. Cells were infected at specified multiplicity of infection (MOI) of influenza virus, based on 6 h focus forming assay (FFA), for 2 h in infection media, after which they were rinsed three times with PBS and incubated in fresh infection media for a further 22 h. MOI of 1.0 is the minimum volume of virus needed to infect all MDCK cells in a culture well. Quantification of infectious virus in spun culture supernatants by FFA was conducted as previously described [18], which was an immuno-cytochemical assay based on infection of MDCK cells with harvested supernatants for 6 h followed by immunodetection of viral nucleoprotein (NP). Briefly, MDCK cells infected for 6 h were fixed in acetone methanol for 10 min followed by peroxidase treatment for 10 min and incubation with a 1:8000 dilution of primary mouse monoclonal antibody to influenza nucleoprotein (Abcam, ab20343, Cambridge, UK) for 40 min at room temperature. The cells were subsequently rinsed with Tris-buffered saline (TBS), incubated with horse radish peroxidase-labelled polymer for 40 min. After rinsing with TBS, the cells were incubated with DAB substrate-chromogen solution for 7 min (Envision+ system-HRP kit, Dako, Glostrup, Denmark). Cells positive for viral nucleoprotein were counted with an inverted microscope, and the mean of positive cells in five 96-wells was used to calculate infectious focus-forming units of virus per microlitre of infection volume. H3N8 progeny virus output was determined by TCID_50_ assay, based on 10-fold diluted spun sns to infect MDCK cells for 48 h in a 96-well format followed by hemagglutination and results presented as log_10_(TCID_50/mL_), based on Reed and Muench (1938) [19].

### 2.5. RNA Preparation and Real-Time RT-PCR

Total RNA was extracted from cells using the RNeasy Plus Minikit (Qiagen, Hilden, Germany). Viral RNA in the spun culture supernatants was extracted with the QIAamp Viral RNA Mini Kit (Qiagen). cDNA was synthesised from 0.5 or 1 µg of total RNA using Superscript III First Strand synthesis kit (Thermo Fisher Scientific). Expression of host genes was performed with a LightCycler−96 instrument (Roche, Basel, Switzerland), and computation was based on the comparative Ct approach, normalised to *18S* ribosomal RNA. Human ER stress primers for *HSPA5* (FH1_HSPA5 and RH1_HSPA5), *HSP90B1* (FH1_HSP90B1 and RH1_HSP90B1) and *DDIT3* (FH1_DDIT3 and RH1-DDIT3), human *OAS1* primers (FH1_OAS1 and RH1_OAS1), and human *IFNB1* (FH1_IFNB1 and RH1_IFNB1) were pre-made designs from Sigma-Aldrich. Primers (synthesised by Sigma-Aldrich) for USSR H1N1 M-gene, human *RIG-I* (*DDX58*), *IFNL2* and *IFNL3* for pig *RIG-I* and *OAS1* were designed using PrimerExpress 3.0.1 (Thermo Fisher Scientific); sequences described in Table 1. Human *IRF9* and *OAS3*, and pig ER stress primers for *HSPA5*, *HSP90B1* and *DDIT3* were pre-made designs from Sigma-Aldrich.

### 2.6. Western Blotting

Cells were lysed by radioimmunoprecipitation assay (RIPA) buffer (Santa Cruz, Dallas, TX, USA) supplemented with 1% phenylmethylsulfonyl fluoride (PMSF) (Santa Cruz), 1% inhibitor cocktail, and 1% sodium orthovanadate, according to manufacturer’s instructions (Santa Cruz). Protein concentration was determined by Bio-RAD protein assay (Bio-Rad, Hercules, CA, USA). Primary antibodies were goat antiviral M1 at 1:500 dilution (Abcam, ab20910), rabbit antiviral NP at 1:500 dilution (Thermo Fisher Scientific, PA5-32242) and mouse anti-β-actin at 1:10,000 (Sigma-Aldrich, A5316), and appropriate species-specific secondary antibodies were peroxidase-conjugated for chemiluminescence detection (Amersham ECL Western Blotting Detection Reagent, Marlborough, MA, USA).

### 2.7. Confocal microscopy

NHBE cells were cultured in an eight well µ-slide (ibidi) and at ~70% confluence primed with 0.01 μM TG or DMSO for 30 min. Cells were washed with PBS and infected with USSR H1N1 virus at 1.0 MOI for 6 h; they were then washed twice with PBS and fixed in fresh 4% paraformaldehyde in PBS, adjusted to pH 7.4, for 20 min at room temperature. The cells were permeabilised with 0.1% Triton X-100 (Sigma-Aldrich) for 5 min at 4 °C. After washing with PBS, the samples were incubated in PBS supplemented with 3% BSA and 1% glycine for 30 min in a humidified chamber at room temperature, washed twice with PBS (5 min each time) and incubated under the same conditions in 10% serum with a secondary antibody donor, diluted in PBS. The samples were incubated with the primary antibody anti-Influenza A Virus nucleoprotein antibody (AA5H) at 6 μg/mL, Abcam) diluted in 10% serum in PBS, overnight at 4 °C. The samples were washed with PBS and the secondary antibody (anti-mouse IgG H&L Alexa Fluor^®^ 488, Thermo Fisher Scientific) at 2 μg/mL, diluted in 10% serum in PBS, was added for 1 h at room temperature in a humidified chamber. The secondary antibody was removed and the cells thoroughly washed with PBS. After immunostaining for all of the proteins of interest, the cells were washed thoroughly with PBS. ProLong™ Gold Antifade Mountant with DAPI (Invitrogen) was added to each well at a sufficient volume to cover the base of the well and incubated at room temperature for 24 h. The NHBE cells were subsequently stored at 4 °C until imaging with a Leica TCS SP8 confocal microscope using a 63-time oil immersion objective. Integrated density was measured using Image J 1.x [20].

### 2.8. Influenza Virus Challenge in Mice

To assess the antiviral function of TG in vivo, 6- to 8-week-old BALB/c mice (female) were organised into three separate groupings to determine: post-infection survival (*n* = 10 for each of 2 groups), progeny virus production (*n* = 6 for each of 2 groups), and changes in immuno-histopathology (*n* = 6 for each of 2 groups). Each mouse was treated with 30 ng of TG or control PBS-DMSO (percentage DMSO in PBS-DMSO and TG was the same) by gavage 12 h prior to intranasal infection with 1 × 10^2^ TCID_50_ of PR8 virus (~4 times LD_50_ dose) PBS at 50 μL or with mock PBS control as previously described [21]. The dose of TG selected was based on a preliminary in vivo study and was well tolerated by the mice. Subsequently, mice were given the same daily dose of TG or PBS-DMSO by gavage until 7 days dpi. Mouse survival and body weight were monitored daily. Lungs of three mice from each TG treated group and three from each PBS-DMSO group were collected 3 dpi, and again 5 dpi, for viral titre determination and immuno-histopathology. Virus titration was performed by TCID_50_ assays on MDCK cells inoculated with 10-fold serially diluted homogenised lung tissues and incubated at 37 °C in a 5% CO2 atmosphere for 72 h. TCID_50_ values were calculated according to the Reed-Muench method [19]. Lung sections were incubated with anti-nucleoprotein (NP) antibody (1:100 dilution; Abcam ab20343) at 4 °C overnight in a humidified chamber, then incubated with horseradish peroxidase-conjugated secondary antibody for 60 min at room temperature. Signal was detected using the Vector Elite ABC Kit (Vectastain, Vector Laboratories, Burlingame, CA, USA). Lung sections were also stained with hematoxylin and eosin.

### 2.9. RNA-Sequencing

At greater than 80% confluence, NHBE cells were primed with 0.01 µM TG or DMSO control for 30 min, washed with PBS and infected with USSR H1N1 virus at 0.8 MOI for 12 h. Total RNA extraction was performed using the RNeasy Plus Mini Kit (Qiagen) and ribosomal RNA depletion was applied. For each treatment condition, a total of four samples were used for RNA-seq, comprising two technical replicates of two biological replicates (from two donors). TruSeq Stranded Total RNA libraries were generated using ribosomal-depleted total RNA and sequenced using a HiSeq 3000 System (SCIF NGS Facility). Adaptor sequences were removed using skewer v0.2.2. Reads were filtered to have average quality score of 20 or greater, and the 3′ ends were filtered to have a quality score of 3 or greater. The human transcriptome, containing human mRNAs and non-coding RNAs, was downloaded from Ensembl 92 (GRCh38.p12). Influenza virus sequences were downloaded from the OpenFlu database for the OpenFlu Isolate IDOFL_ISL_21018 (A/USSR/90/1977), last updated 2016-11-30. Sequence alignment and read quantification was performed using the pseudo-alignment-based tool Kallisto v0.43.0 [22]. Differential expression was determined using Sleuth v0.28.1 [23]. Differentially expressed genes were analysed using the KEGG pathways database [24] described via NIPA (version 0.6.5) (available from https://github.com/ADAC-UoN/NIPA), and by Ingenuity pathway analysis (IPA, Qiagen). The RNA-seq data generated during this study are available at GEO (GSE120730).

### 2.10. Quantification and Statistical Analysis

Statistical analysis was performed using GraphPad Prism 7 and the statistical method used described in the figure legend. *p*-value < 0.05 was considered significant, * *p* < 0.05, ** *p* < 0.01, *** *p* < 0.001, **** *p* < 0.0001. Kaplan-Meier method was employed for survival analysis. For RNA-seq analysis, transcripts with a false detection rate corrected *p*-value < 0.05 and a log_2_-fold change (log_2_FC) greater than 1 and less than minus 1 were considered to be differentially expressed, unless otherwise stated. False detection rate correction was performed using the Benjamini-Hochberg method [25]. Results presented representative of two or more independent repeats and all error bars are standard deviations.

### 2.11. Ethics Statement

All animal work was approved by the Beijing Association for Science and Technology (approval ID SYXK (Beijing) 2007-0023; approval date: 30 August 2020) and conducted in accordance with the Beijing Laboratory Animal Welfare and Ethics guidelines, as issued by the Beijing Administration Committee of Laboratory Animals, and in accordance with the China Agricultural University Institutional Animal Care and Use Committee guidelines (ID: SKLAB-B-2010-003, approval date: 30 August 2020).

## 3. Results

### 3.1. Non-Cytotoxic Application of TG Blocks Influenza A Virus Replication in Respiratory Epithelial Cells

**Figure 1:**
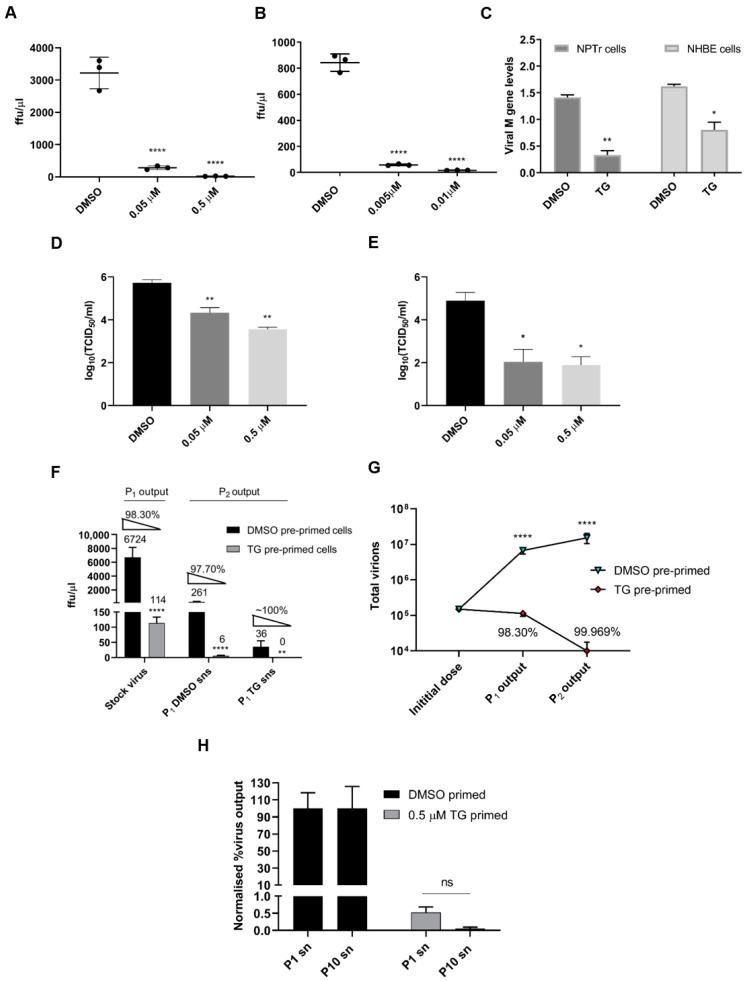
Brief TG priming of NPTr and NHBE cells blocks progeny influenza virus production. (**A**) NPTr cells and (**B**) NHBE cells were incubated for 30 min with TG or DMSO control at the indicated concentrations, PBS washed and infected for 12 and 24 h, respectively, with USSR H1N1 virus at 0.5 MOI. Spun supernatants (sns) were used to infect MDCK cells for 6 h in FFAs. Significance determined by one-way ANOVA, relative to DMSO control. (**C**) NPTr cells and NHBE cells were primed with TG (0.5 and 0.01 µM, respectively) or DMSO for 30 min, and infected with USSR H1N1 virus (0.5 and 1.0 MOI, respectively) for 24 h. Culture media were then harvested to determine presence of viral M-gene RNA. Significance determined by paired *t*-test. (**D**,**E**) MDCK cells were incubated for 30 min with TG or DMSO control at the indicated concentrations, PBS washed and infected for 24 h with (**D**) equine H3N8 and (**E**) canine H3N8 virus. TCID50 assay was performed to quantify virus output. Significance determined by one-way ANOVA, relative to DMSO control. (**F**) P_1_ sns were derived from 0.5 µM TG or DMSO pre-primed (for 30 min) NPTr cells infected with stock virus (USSR H1N1 at 0.5 MOI) for 23 h. Fresh NPTr cells (P_2_) pre-primed with respective TG or DMSO were infected with corresponding P_1_ sns for 23 h. Volume of P_1_ supernatants used was the same as the starting stock virus volume. Significance based on mixed model analysis was relative to corresponding DMSO pre-primed cells. (**G**) Total progeny virus yield from initial virus infection on P_1_ cells, pre-primed with DMSO or TG, followed by sequential infection with P_1_ supernatants on P_2_ cells correspondingly pre-primed with DMSO or TG. Significance based on mixed effect analysis was relative to corresponding DMSO pre-primed cells. Log (total virus yield) displayed. Each % refers to reduction in virus output relative to initial virus dose from TG priming. (**H**) At passage 1 (P_1_), cells were infected with 20 HA units of USSR H1N1 virus for 2 h, PBS washed and cultured for 3 days in the continuous presence of 0.05 µM TG or DMSO control in serum-free OptiMEM media, supplemented with TPCK trypsin at 0.2 µg/mL. Spun P_1_ sns were used for the next passage; a total of 10 serial passages were completed. P_1_ and P_10_ sns were subsequently used in duplicates to separately infect for 2 h NPTr cells, pre-infection primed for 30 min with 0.5 µM TG or DMSO, washed with PBS and cultured in OptiMEM for a further 22 h, after which harvested sns were used in 6 h FFAs on MDCK cells to determine progeny virus output expressed in percentage where corresponding DMSO control output was set at 100%. Two-way ANOVA, Sidak’s multiple comparisons test, showed no significance (ns) in virus inhibition between P_1_ and P_10_ TG-derived sns. Significance determined by Mann Whitney test, relative to corresponding DMSO control. * *p* <0.05, ** *p* <0.01, **** *p* <0.0001. All assays were in triplicates and were performed three times.

To ascertain an innate immune role for TG on influenza A virus replication, NPTr cells, a neonatal porcine tracheal epithelial cell line [26] (Figure 1A) and primary normal human bronchial epithelial (NHBE) cells (Figure 1B) were primed with TG for a total duration 30 min and then infected with a human USSR H1N1 virus. TG priming dramatically reduced progeny virus production 12 and 24 h post-infection as determined by focus forming assays (FFAs) (Figure 1A,B). Viral M-gene RNA in the culture media of TG-primed cells was also reduced relative to control cells, indicating an overall reduction in progeny virus release from TG-primed cells (Figure 1C). TG priming was effective against other influenza A subtypes, reducing progeny virus output in equine and canine H3N8 viruses in MDCK cells (Figure 1D,E). Moreover, one passage of supernatants (sns) derived from initially infected TG-primed NPTr cells (P_1_) to fresh TG-primed cells (P_2_) almost completely abolished progeny virus production to around the threshold of viral detection, highlighting the highly potent antiviral effect of TG (Figure 1F,G). Moreover, USSR H1N1 virus was subjected to 10 successive passages in NPTr cells in the continuous presence of DMSO or sub-optimal TG (0.05 µM). The sensitivity of the first (P_1_) and 10th passaged virus (P_10_) to TG (30 min priming at 0.5 µM) remained unchanged as indicated by no increase in progeny output after 10 passages (Figure 1H).

**Figure 2:**
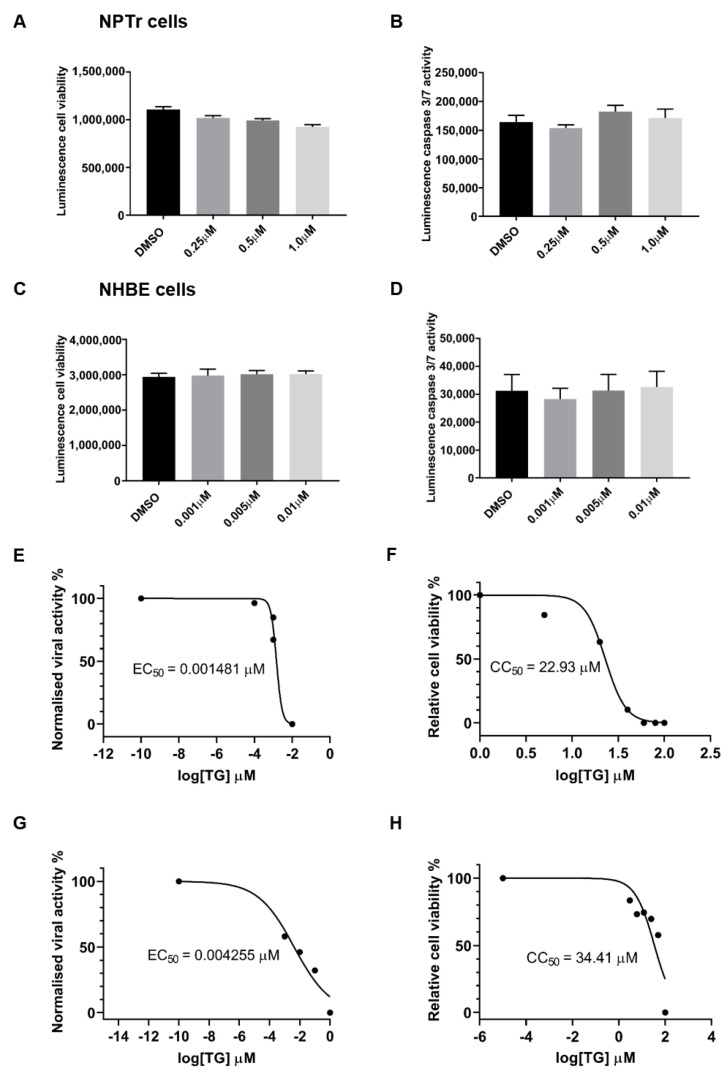
Priming with antiviral concentrations of TG displays no cytotoxicity in respiratory epithelial cells. (**A**,**B**) NPTr cells and (**C**,**D**) NHBE cells were incubated in indicated concentrations of TG or in DMSO control for 30 min and (**A**,**C**) cell viability assays (CellTiter-Glo luminescent cell viability assay) or (**B**,**D**) caspase 3/7 activity assays (Caspase-Glo 3/7 assay) performed 24 h later. Significance determined by one-way ANOVA, relative to DMSO control. No significant change in cell viability or caspase activity was found. (**E**) NHBE cells and (**G**) NPTr cells were incubated for 30 min in a range of TG concentrations, PBS washed and infected for 24 h with USSR H1N1 virus at 1.0 MOI and 0.5 MOI, respectively. Spun sns were used to infect MDCK cells for 6 h in FFAs. EC_50_ was calculated from progeny virus output (%) by non-linear regression. (**F**) NHBE cells and (**H**) NPTr cells were incubated for 30 min in a range of TG concentrations, PBS washed and cell viability assay (CellTiter-Glo luminescent cell viability assay) performed 24 h later. CC_50_ was calculated from relative cell viability (%) by non-linear regression. SIs (CC_50_/EC_50_) for NHBE and NPTr cells are 15,483 and 8087, respectively.

The antiviral doses of TG used to prime NPTr and NHBE cells had no significant adverse effect on cell viability, based on host ATP production (Figure 2A,C), and caspase 3/7 activity (Figure 2B,D). The selectivity index (SI) of TG in virus inhibition in NHBE cells and NPTr cells was similarly high. SI from 30 min of pre-infection TG priming of NHBE cells was 15,483 (cytotoxicity concentration_50_ (CC_50_) (based on luminescence cell viability) = 22.93 μM, and inhibitory concentration_50_ (EC_50_) (based on virus output, ffu/µL) = 0.001481 μM), and in NPTr cells was 8087 (CC_50_ = 34.41 μM, and EC_50_ = 0.004255 μM) (Figure 2E–H).

**Figure 3:**
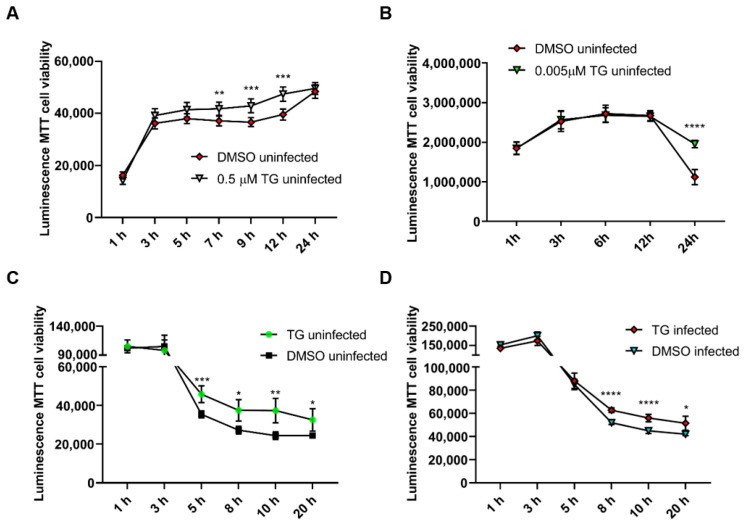
Continuous exposure of uninfected cells to antiviral doses of TG does not affect cell viability. Cell viability of (**A**) NPTr cells continuously incubated in the presence of 0.5 μM TG and (**B**) NHBE cells continuously incubated with 5 nM TG was monitored over 24 h with RealTime-Glo™ MT Cell Viability Assay. Readings indicate duration of TG exposure. Significance based on mixed effect analysis relative to corresponding DMSO control. Cell viability of (**C**) uninfected NPTr cells and (**D**) NPTr cells infected with 0.5 MOI USSR H1N1, pre-primed with 0.5 μM TG for 30 min, was monitored with RealTime-Glo™ MT Cell Viability Assay. Readings indicate duration of post mock or influenza virus infection. Significance based on mixed effect analysis relative to corresponding DMSO control. * *p* < 0.05, ** *p* < 0.01, *** *p* < 0.001, **** *p* < 0.0001.

Furthermore, continuous exposure of NPTr cells and NHBE cells to antiviral doses of TG (at 0.5 μM and 0.005 μM, respectively) for 24 h not only showed no cytotoxic effect but appeared to confer some improvement in cell viability during culture (Figure 3A,B). Pre-priming with 0.5 μM TG for only 30 min did not reduce cell viability in uninfected or influenza virus-infected NPTr cells but showed improved viability during the 20 h culture (Figure 3C,D).

Potential TG cytotoxicity was also evaluated in RNA-seq analysis (see Section 3.4), utilising Ingenuity Pathway Analysis (IPA)-Tox, a capability tool within the IPA knowledge base, to predict drug mechanisms of toxicity in uninfected or infected (0.8 MOI USSR H1N1, 12 h infection) NHBE cells. In uninfected NHBE cells, TG priming (0.01 μM, 30 min) was not associated with any significant z-score, describing the activation state of a biological function. In infected NHBE cells, the only significant z-score associated with TG priming predicted decreased liver damage (z-score −2.2, overlap *p*-value 0.499, thereby providing further support for the non-cytotoxic nature of TG doses used to induce an antiviral state. In summary, brief priming of cells with non-cytotoxic concentrations of TG strongly inhibits influenza virus replication.

### 3.2. TG Promptly Induces an Extended Antiviral State Targeting Influenza Virus Post-Translationally

**Figure 4:**
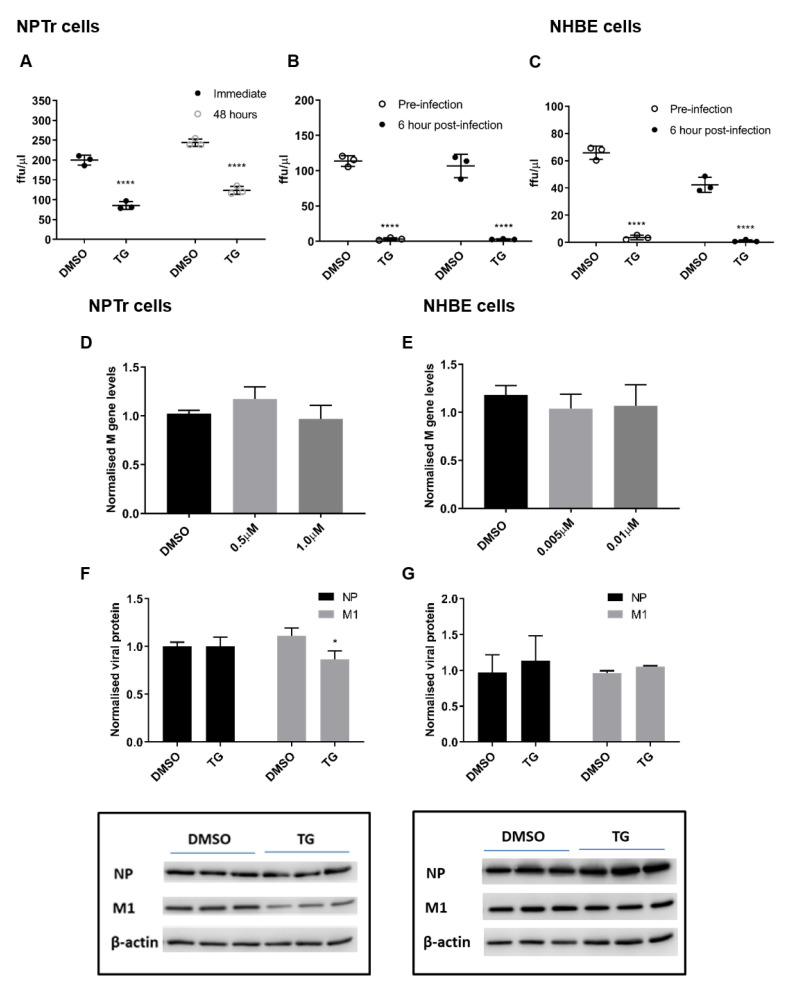
TG promptly induces an extended antiviral state targeting influenza virus post-translationally. (**A**) NPTr cells primed with 0.5 µM for 30 min were immediately infected or further cultured for 48 h prior to infection with USSR H1N1 virus at 0.5 MOI for 24 h. Spun sns were used to infect MDCK cells for 6 h in FFAs. Significance determined by paired t-test within each time point. (**B**) NPTr and (**C**) NHBE cells were incubated with TG (0.5 and 0.01 µM, respectively) for 30 min and immediately infected (TG pre-infection) or were first infected for 6 h followed by TG exposure for 30 min (TG post-infection). NPTr and NHBE cells were infected with USSR H1N1 virus at 0.5 and 1.0 MOI, respectively. TG pre-primed cells were infected for 2 h, PBS washed and cultured in fresh infection media overnight. Cells 6 h post-infection were treated with TG for 30 min, washed with PBS and cultured in fresh infection media overnight. Spun sns were used to infect MDCK cells for 6 h in FFAs. Significance determined by paired t-test within each time point. (**D**,**F**) NPTr primed with (0.5 or 1.0 µM) and (**E**,**G**) NHBE cells primed with TG (0.005 or 0.01 µM respectively) were subsequently infected with USSR H1N1 at 0.5 and 1.0 MOI respectively for 24 h. (**D**,**E**) Total RNA extracted to determine M gene expression for all samples, normalised to 18S rRNA. Significance determined by one-way ANOVA, relative to DMSO control. (**F**,**G**) Protein lysates (DMSO and highest selected TG concentration) harvested at 24 h post-infection were used to ascertain viral NP and M1 protein levels, normalised to beta-actin. Representative blots displayed. Significance determined by paired t-test. * *p* < 0.05, **** *p* < 0.0001. All assays were in triplicates and were performed three times.

The duration of virus inhibition in NPTr cells from 30 min of TG pre-infection priming was ound to last at least 48 h (Figure 4A). Furthermore, virus inhibition was similarly effective between pre-infection and 6 h post-infection TG primed NPTr (Figure 4B), and NHBE cells (Figure 4C) (virus replication cycle is around 6 h). Inhibition of progeny virus production in TG-primed NPTr and NHBE cells was accompanied by little or no change in viral RNA (Figure 4D,E) and viral proteins (Figure 4F,G). Collectively, the lack of substantive change in the expression of viral RNA and protein in infected cells pre-primed with TG indicates that a key virus inhibition step occurred post-translationally, possibly affecting viral protein processing or transport.

**Figure 5:**
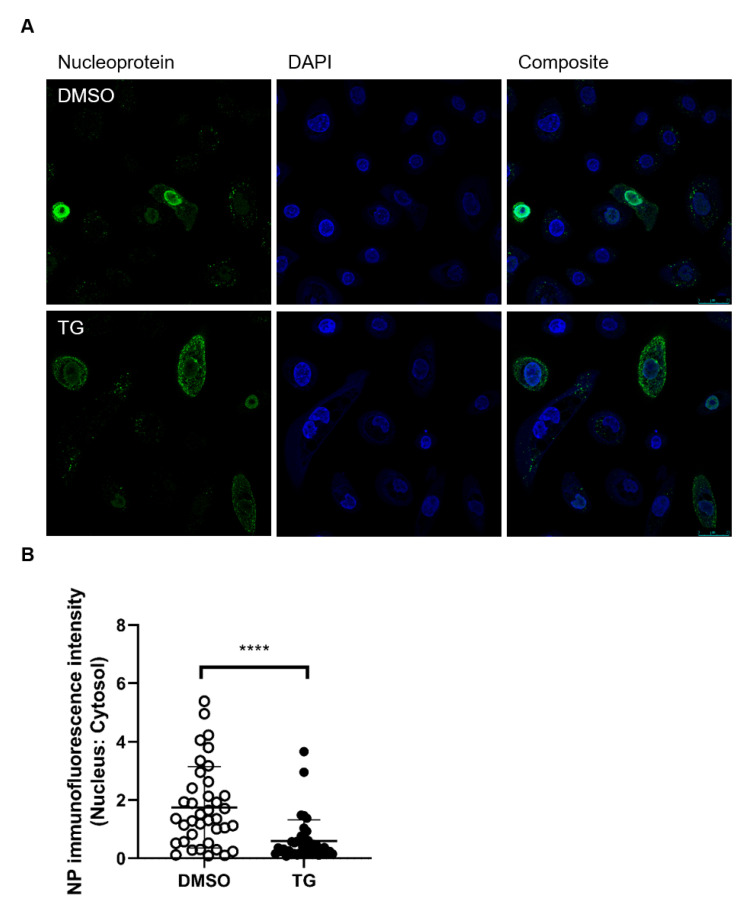
Cytoplasmic NP localisation in TG primed cells. (**A**) NHBE cells were primed with DMSO or 0.01 μM TG for 30 min, and infected with USSR H1N1 virus at 1.0 MOI for 6 h. Cells were immunostained for viral NP (mouse anti-influenza A virus NP primary antibody detected by donkey anti-mouse IgG H&L conjugated to Alexa Fluor 488 secondary antibody; green fluorescence) and nuclei were stained with DAPI (blue fluorescence). Cells imaged with a Leica TCS SP8 confocal microscope using a 63-time oil immersion objective (scale bar: 25 µm). (**B**) The ratio of nuclear to cytoplasmic NP fluorescence signal in DMSO and TG-primed NHBE cells was calculated using Fiji (image J). Significance determined by Mann Whitney test, relative to indicated DMSO control. **** *p* < 0.0001.

Viral NP localisation in TG-primed NHBE cells was performed at 6 h post-infection (hpi). Infected TG-primed NHBE cells exhibited reduced nuclear accumulation of viral NP; there was more than three-fold reduction in mean nuclear to cytosolic localisation ratio in TG treated cells (Figure 5), which suggests that transport of NP into the nucleus was impaired by TG priming. Thus, brief TG priming promptly confers an extended antiviral state of at least 48 h and disrupts viral NP protein trafficking.

### 3.3. TG in Mice Confers Protection Against Lethal Virus Challenge

**Figure 6:**
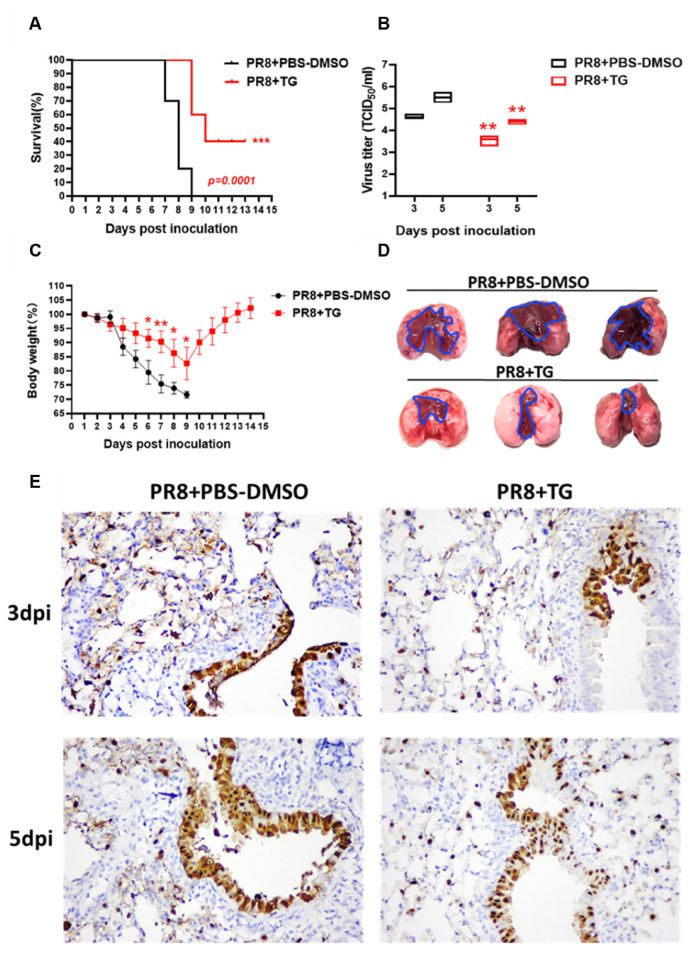
TG in mice confers protection against lethal PR8 H1N1 virus challenge. (**A**) Survival of mice post-infection treated each day with TG or PBS-DMSO control (*n* = 10 in each group). Kaplan-Meier survival curves are compared using the log-rank (Mantel-Cox) analysis. (**B**) Viral titres of lungs from mice treated with TG or PBS-DMSO at 3dpi and 5dpi was determined by TCID_50_ assays (*n* = 3 in each group). (**C**) Mean body weight changes post-infection was determined by daily monitoring (*n* = 10 in each group). (**D**) At 5 dpi, entire lungs of TG treated mice displayed much less extensive gross pathology than those of control lungs. Blue outline demarcates boundary between apparent normal and consolidated abnormal tissues. (**E**) Representative microscopic lung fields, (40-time magnification), derived from similar anatomical sites taken at 3 and 5 dpi, showed that TG treatment resulted in less diffused distribution of viral NP protein (less brown staining), and more frequent localisation of NP than those in corresponding PBS-DMSO controls. Each mouse was infected at 1 × 10^2^ TCID_50_ of PR8 virus. ** *p* < 0.01, *** *p* < 0.001.

As proof of principle for the antiviral use of TG in vivo, we examined its effects in mice subjected to lethal PR8 H1N1 virus challenge. We found that mice treated with TG (30 ng) orally (by gavage), before infection and once a day post-infection until 7 days post-infection (dpi), showed improved survival (Figure 6A) (*p* = 0.0001). PR8 virus loads in lungs of TG treated mice were significantly lower than those of corresponding control mice on 3 dpi and 5 dpi (Figure 6B). The 30 ng/mouse/day (1.5 μg/kg/day) dose was a small fraction of the reportedly safe oral (at ~97.62 μg/kg in PCT WO2003/049717) or parenteral (2500 μg/kg) [27] use of TG for other types of in vivo studies. All PBS-DMSO control mice infected with PR8 H1N1 virus died by 9 days dpi whereas 4 out of 10 (40%) TG treated mice survived and showed progressive weight gain from 9 dpi, which also indicates that the TG regime was not detrimental to growth (Figure 6A,C). Significant divergence in mean body weight between TG treated and control mice was evident from 6 dpi (Figure 6C). It should be noted that the antiviral dose of TG at 1.5 μg/kg/day had not been optimised in mice.

Gross difference in lung pathology between TG treated and control mice was clearly visible at 5 dpi. Control lungs showed extensive contiguous hyperaemic consolidation, whereas lung lesions in TG mice were more localised and restricted to the vicinity of virus entry site, indicating reduced virus spread (Figure 6D). Less severe lung pathology seen in TG treated mice coincided with noticeably less cytosolic presence of NP at 3 dpi and 5 dpi than corresponding controls (Figure 6E). Thus, oral administration of TG in mice significantly reduced severity and virus shedding, and improved survival during lethal influenza virus infection.

### 3.4. ER Stress Is a Dominant Driver of Host Antiviral Response to TG Priming

**Figure 7:**
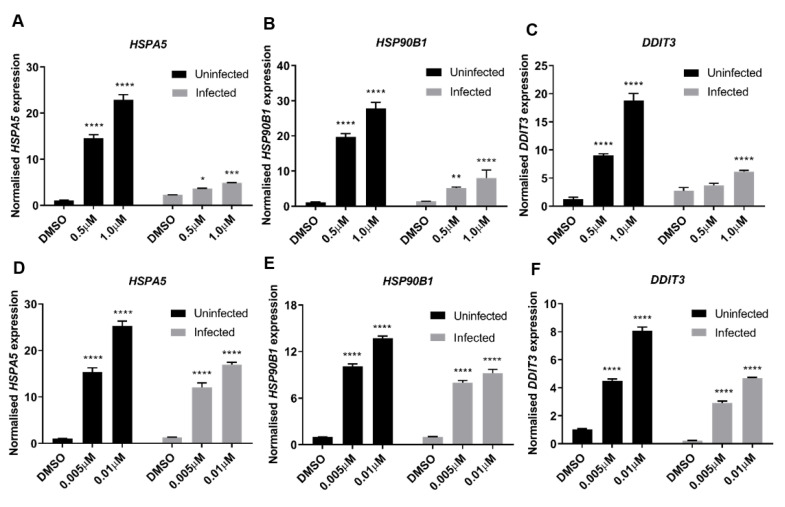
ER stress is a driver of host antiviral response to TG priming. (**A**–**C**) NPTr cells or (**D**–**F**) NHBE cells were primed with DMSO or TG for 30 min at the indicated concentrations and infected for 24 h with USSR H1N1 virus at 0.5 MOI. Total RNA was extracted from each sample for HSPA5, HSP90B1 and DDIT3 detection, normalised to 18S rRNA. Indicated significance based 2-way RM ANOVA relative to corresponding DMSO control. *p < 0.05 ** *p* < 0.01, ***p < 0.001 **** *p* < 0.0001.

RNA-seq analysis, along with analytical use of IPA knowledge base, was performed to identify antiviral processes elicited by TG pre-priming (0.01 μM, 30 min) of NHBE cells infected with 0.8 MOI USSR H1N1 virus for 12 h. We found that the antiviral effects of TG are strongly linked to ER stress-induced UPR [9,15]; it was the most highly enriched canonical pathway upregulated by TG in uninfected cells (−log(*p*-value) 1.87 × 10, ratio 0.286), and infected cells (−log(*p*-value) 1.42 × 10, ratio 0.286). In the absence of TG priming, between uninfected cells and infected cells primed with DMSO, no genes associated with the canonical UPR pathway were significantly differentially expressed due to infection, as can be examined in the full RNA-seq data set deposited in the GEO database (GSE120730). UPR can promote host innate immune response and interfere with viral protein translation [11,28,29]. We corroborated by qPCR that priming with non-toxic doses of TG strongly elevated the basal expression of ER stress-associated genes (*HSPA5*, *HSP90B1,* and *DDIT3*) in NPTr cells (Figure 7A–C) and NHBE cells (Figure 7D–F) in a dose-dependent manner.

**Figure 8:**
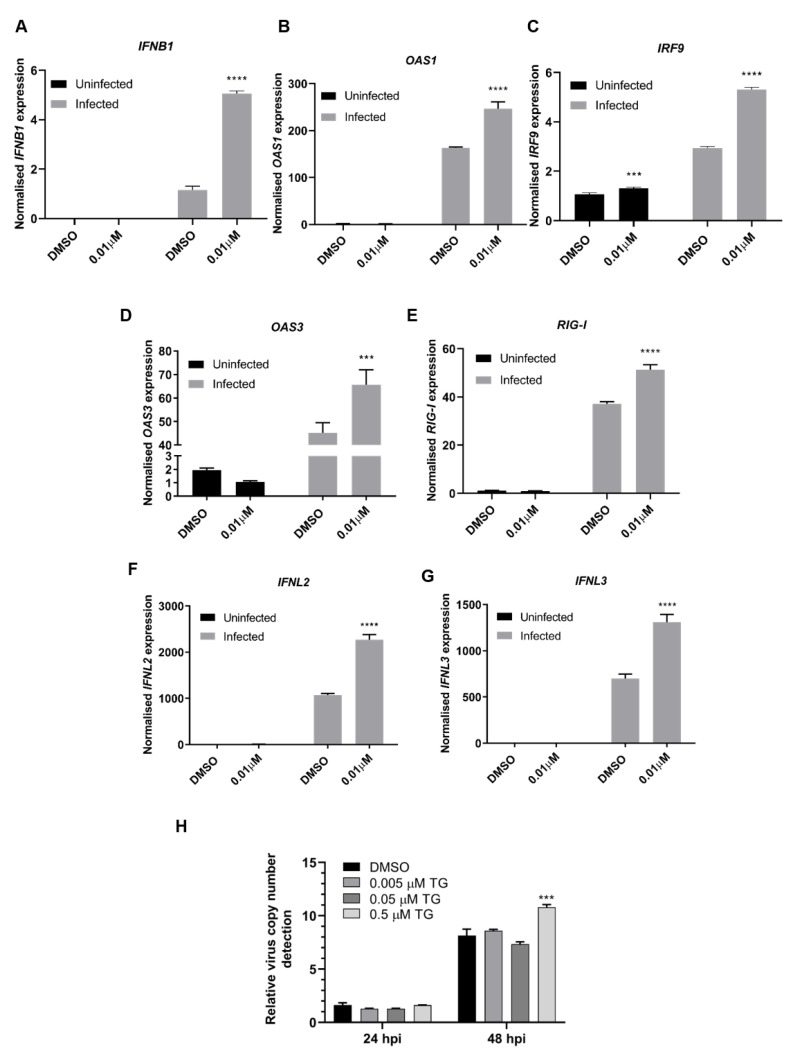
TG priming enhanced type I/III IFN-dependent gene expression. (**A**–**G**) NHBE cells were incubated for 30 min with DMSO or 0.01 µM TG and subsequently infected with USSR H1N1 virus at 0.5 MOI for 24 h. Total RNA was extracted from each sample for type I/III IFN and indicated associated gene expression, normalised to 18S rRNA. Significance determined by two-way ANOVA, relative to corresponding DMSO control. (**H**) Vero cells were primed with TG as indicated for 30 min, washed twice with PBS and infected with USSR virus 0.5 MOI. Viral RNA extraction was performed on culture media at 24 and 48 hpi followed by one-step reverse transcription qPCR to detect the relative copy number of M-gene RNA, based on relative Ct method. Vero cells are unable to produce type I IFNs, which appear necessary for TG to induce an antiviral state. Indicated significance based 2-way RM ANOVA relative to corresponding DMSO control. *** *p* < 0.001, **** *p* < 0.0001.

Type I IFNs and their associated genes are essential for host defence against viruses [30] and can be induced by ER stress [14]. RNA-seq analysis found over 80% of differentially expressed transcripts of type I IFN-associated genes (biological process GO term 0060337) were more highly expressed in TG-primed and infected NHBE cells than those from corresponding DMSO treated and infected controls. This was exemplified by qPCR based on TG primed (0.01 µM, 30 min) NHBE cells infected for 24 h with USSR H1N1 virus. Priming with TG consistently enhanced the expression of the type I *IFNβ1*, interferon regulatory factor 9 (*IRF9*), a factor that transcriptionally activates type I IFN, type I IFN-dependent retinoic acid-inducible gene 1 (*RIG-I*), and IFN-inducible 2′−5′-oligoadenylate synthetase 1 (*OAS1*) and *OAS3* genes (Figure 8A−E). Additionally, expression of type III IFNs (*IFNL2* and *IFNL3)*, was enhanced in TG-primed and infected NHBE cells (Figure 8F,G). The contribution of type I IFN to TG mediated antiviral activity was assessed in Vero cells, a cell type unable to produce type I IFNs [31,32]. TG primed Vero cells did not significantly inhibit USSR H1N1 virus replication, as indicated by viral copy number in culture media, at 24 and 48 hpi (Figure 8H).

To further assess the role of ER stress in TG-mediated antiviral response, we compared the antiviral effect of tunicamycin, a well-established inducer of ER stress, with TG in NPTr cells. Tunicamycin priming of NPTr cells at non-cytotoxic concentrations (at 0.5 or 1.0 µg/mL for 30 min) significantly reduced progeny virus output (Figure S1A,B). Induction of ER stress-associated genes from tunicamycin priming at 0.5 µg/mL was comparable to the use of TG at 0.5 µM, and at 1.0 µg/mL was approximately 3 to 8.2-fold higher than TG priming (Supplementary Figure S1C,D). Despite higher apparent ER stress, progeny virus output from tunicamycin-primed cells was higher (thus less inhibitory) than TG-primed cells (Figure S1B). This could be attributed to differential activation of ER stress UPR pathways by tunicamycin and TG. Enhanced type I IFN-dependent gene expression induced by TG was not found in tunicamycin-primed NPTr cells (Figure S1E,F). Differential activation of the type I IFN response by TG-dependent and not tunicamycin-mediated ER stress has been previously reported [14]. Expression of ER stress genes in TG, and tunicamycin-primed but not DMSO control cells was attenuated during influenza virus infection, which might have been a virus effect to maintain viral protein production (Figure S1C,D). Thus, ER stress *per se* contributes to the antiviral effects of TG priming.

**Figure 9:**
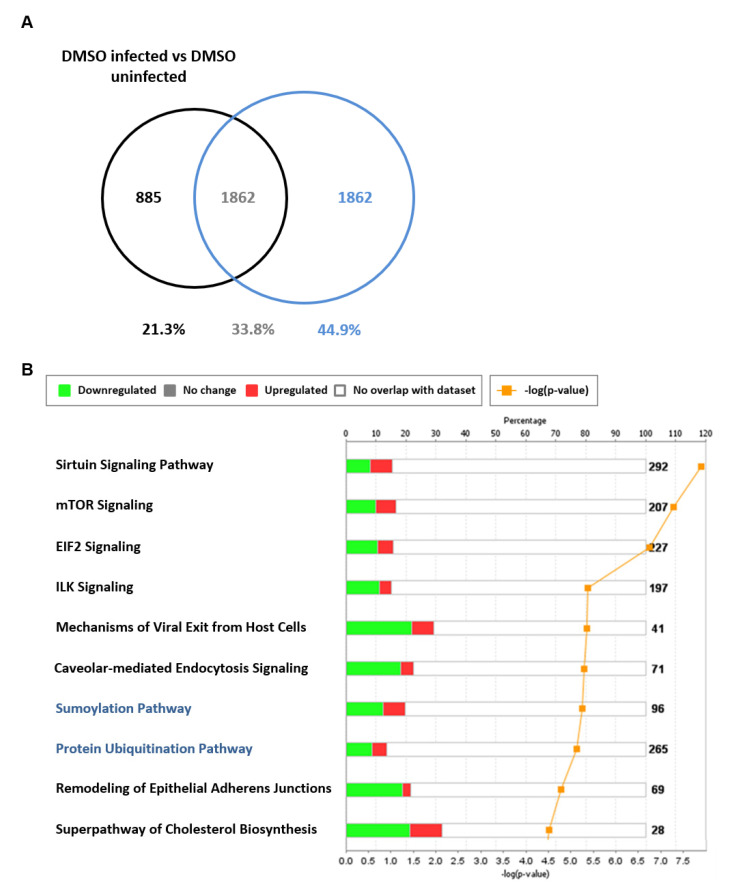
Differentially expressed genes exclusive to TG-primed and infected NHBE cells. NHBE cells were primed with 0.01 µM TG for 30 min, washed with PBS and infected with USSR H1N1 virus at 0.8 MOI for 12 h for RNA-seq followed by IPA analysis. (**A**) Differentially expressed genes (1862 transcripts) in NHBE cells in response to infection (based on filtering parameters of *q* < 0.05 and log2-fold-change < −1 and > +1) were identified as exclusive to TG-primed and infected NHBE cells (i.e., [TG-primed infected/TG-primed uninfected] less (DMSO infected/DMSO uninfected)). (**B**) Protein sumoylation and ubiquitination (highlighted blue) were among the enriched canonical pathways represented in the 1862 transcripts (based on filtering parameters of *q* < 0.05 and log2-fold-change < −1 and > +1) exclusive to TG-primed and infected NHBE cells. The stacked bar chart displays the percentage of significant differentially expressed genes that are upregulated (red) or downregulated (green) in each canonical pathway. The number of genes within the pathway recognised by IPA indicated by the numbers to the right of the bars. Orange line represents the −log *p*-value, indicating the statistical significance of the over-represented canonical pathway.

Additional host antiviral processes induced by TG priming were further evident in the analysis of 1862 transcripts (encoded by 1468 genes) exclusively found in the differential comparison of TG-primed infected vs. TG-primed uninfected NHBE cells (Figure 9A). This data set did not include genes differentially expressed in infected DMSO versus uninfected DMSO treated controls. Using IPA, a subset of 87 genes from the 1468 genes were identified as connected to RNA virus replication, 61 of which were predicted, based on direction of differential expression, to reduce RNA virus replication (z = −4.22, overlap *p*-value 2.36 × 10^−11^). Forty-three out of the same 87 genes were specifically associated with influenza A virus replication of which 31 were predicted by IPA to specifically reduce influenza A replication (z = −2.813, overlap *p*-value 9.82 × 10^−6^). The 61 differentially expressed antiviral genes exclusive to TG priming and infection could be further classified into several functional categories. Twenty-one genes (34%) were involved in disruption of protein transport (nuclear, cytoplasmic, secretory or budding); 11 genes (18%) were related to reduced ubiquitinase or kinase function; 8 genes (13%) were associated with enhanced type I/III IFN-associated response; and 8 genes were connected to reduced RNA production or processing. Within the group of 1862 transcripts, sumoylation (−log(*p*-value) 5.27, ratio 0.198) and protein ubiquitination (−log(*p*-value) 5.14 ratio 0.136) were the seventh and eighth most enriched canonical pathways identified (Figure 9B). As influenza viral proteins utilise host post-translational modification machinery to support their replication, including sumoylation and ubiquitination [33,34,35], it is likely that post-translational viral protein modification is part of a multifaceted host antiviral response induced by TG.

Taken together, TG-induced ER stress appears as a key driver responsible for a number of host antiviral processes that include an enhanced type I/III IFN response and involve dysregulation of post-translational viral protein modification.

## 4. Discussion

The problem of drug resistance faced by current and near-market antivirals is a serious concern, as exemplified by the alarming emergence of resistance to the recently approved antiviral baloxavir marboxil (Xofluza) [2,36,37]. Targeting the virus proteins directly applies a strong selection pressure for the emergence of drug-resistance escape mutants [38,39]. Host-dependent therapies are a potential alternative to targeting the virus proteins directly and are not readily overcome by the highly mutable influenza virus [5].

We discovered that SERCA inhibitor TG elicits a range of host antiviral defences including an enhanced type I/III IFN response that is highly inhibitory to influenza A virus replication. Brief TG exposure, at non-toxic levels, promptly induces a prolonged (≥48 h) antiviral state that is potentially of prophylactic and therapeutic importance. Ten passages of USSR H1N1 virus in the continuous presence of suboptimal concentration of TG did not reduce viral sensitivity to subsequent antiviral TG priming, suggesting that TG treatment may be less susceptible to drug resistance. Crucially, daily oral dose of TG in mice, which at 30 ng was a fraction of the reported oral (in PCT WO2003/049717) or parenteral [27] doses given to mice, significantly reduced severity and improved survival in lethal influenza virus infection.

TG triggers three closely connected signalling events: (1) ER Ca^2+^ store depletion, (2) ER stress and (3) extracellular Ca^2+^ entry through the store-operated Ca^2+^ channel. Release of intracellular store Ca^2+^ was found to be more necessary than extracellular Ca^2+^ influx in the inhibition of henipavirus replication using a number of Ca^2+^ mobilisation compounds including TG whose antiviral effect was reduced by the Ca^2+^ chelator, BAPTA-AM at 10 µM [40]. An earlier report showed that inhibition of the IRE1 pathway with tauroursodeoxycholic acid blocks influenza A virus replication [12]. On the contrary, ER stress-activated IRE1α was found to promote antiviral RIG-I-IFN-β signalling in FMD virus inhibition [14,15,16]. Likewise, TG treatment of U2OS cells infected with tick-borne encephalitis virus was found to lead to early IFNβ induction via activated IRE1 [29]. In TG-induced UPR, activated PERK by inactivating the eIF2α subunit rapidly attenuates global protein synthesis that includes that of influenza virus protein synthesis [41]. Although TG-induced UPR is a primary antiviral response to influenza virus inhibition, future studies will need to examine the relative importance of each of the three arms of the UPR.

Our data indicate that TG priming elevates type I/III IFN-associated responses in influenza virus-infected cells. A previous report found stimulating an increase in cytosolic Ca^2+^ in the absence of ER stress did not induce phosphorylation of IRF3, and that ER stress involving Ca^2+^ mobilisation, requiring TBK1 and STING, activates IRF3 [14]. Activation of the STING-TBK1-IRF3 signalling pathway can be inhibited by an intracellular Ca^2+^ chelator [42]. Interestingly, ionomycin stimulated extracellular Ca^2+^ influx was found to inhibit STING mediated production of IFNβ [42]. These findings support the suggestion that TG mediated ER stress from ER store Ca^2+^ efflux promotes the activation of type I/III IFN signalling. The necessity of a functional type I IFN response in a TG induced antiviral state was evident by its ineffectiveness in inhibiting influenza virus replication in Vero cells, a monkey kidney epithelial cell line incapable of producing type I IFNs. Promoting an enhanced type I/III IFN-associated response to infection could also be of paracrine importance in converting neighbouring uninfected cells into a highly activated antiviral state, a containment feature that was evident in infected lungs of TG treated mice.

We identified a TG-specific host antiviral signature of 61 genes whose direction of differential expression is predicted to be consistent with reduced RNA virus replication. Just over 50% of the 61 signature genes are involved in post-translational protein processing (including ubiquitination and sumoylation) or transport across different organelles. Sumoylation of influenza viral proteins, NS1 [43], NP [33] and M1 [34] is either necessary for or promotes virus replication at post-translational stages involving viral protein stability, trafficking or assembly. Ubiquitinaton of influenza viral polymerase complex stimulates polymerase function [44]; and ubiquitination of influenza virus M2 is crucial for virus assembly and virus induced cell death [35]. Thus, reduced or dysregulated ubiquitination or sumoylation of viral proteins can be expected to interfere with viral protein transport and assembly. Future work will benefit from developing an in-depth knowledge of the impact of TG exposure on the host protein processing machinery. Finally, given the structural complexity of TG, there is a possibility that it could also activate host antiviral processes that are independent of its impact on ER stress.

In summary, we propose that TG, in orchestrating a spectrum of host antiviral defences, are not readily overcome by virus mutation, and is potentially a viable host-centric antiviral for the treatment of influenza A virus infection without the inherent problem of drug resistance.

## 5. Patents

The use of TG and other structurally-related compounds in antiviral therapy is covered by patent PCT/GB2019/050977.

## Figures and Tables

**Table 1:** Primer sequences.

Gene	Sense Primer (5′–3′)	Antisense Primer (5′–3′)
*18S* ribosomal RNA (universal)	ACGGCTACCACATCCAAGGA	CCAATTACAGGGCCTCGAAA
*RIG-I* (human)	GAAGGCATTGACATTGCACAGT	TGGTTTGGATCATTTTGATGACA
*IFNL2* (human)	GACCCAGCCCTGGTGGAC	GCTGGATACAGGCCCGGAA
*IFNL3* (human)	GACCCAGCCCTGGGGGAT	GCTGGATACAGGCCCGGAG
*RIG-I* (pig)	CCCTGGTTTAGGGACGATGAG	AACAGGAACTGGAGAAAAGTGA
*OAS1* (pig)	GAGCTGCAGCGAGACTTCCT	GGCGGATGAGGCTCTTCA
*M*-gene (USSR H1N1)	AGATGAGCCTTCTAACCGAGGTCG	TGCAAAAACATCTTCAAGTCTCTG

## Supplementary Materials

The following are available online at https://www.mdpi.com/1999-4915/12/10/1093/s1, Figure S1: TG and tunicamycin mediated ER stress.
