# Supplementary material for: Thapsigargin at Non-Cytotoxic Levels Induces a Potent Host Antiviral Response that Blocks Influenza A Virus Replication
Source: Viruses. 2020 Sep 27;12(10):1093. doi: 10.3390/v12101093 (PMC7600819; doi:10.3390/v12101093)
Supplement: Supplementary file 1 [file viruses-12-01093-s001.pdf]

viruses

MDPI

Supplementary Material

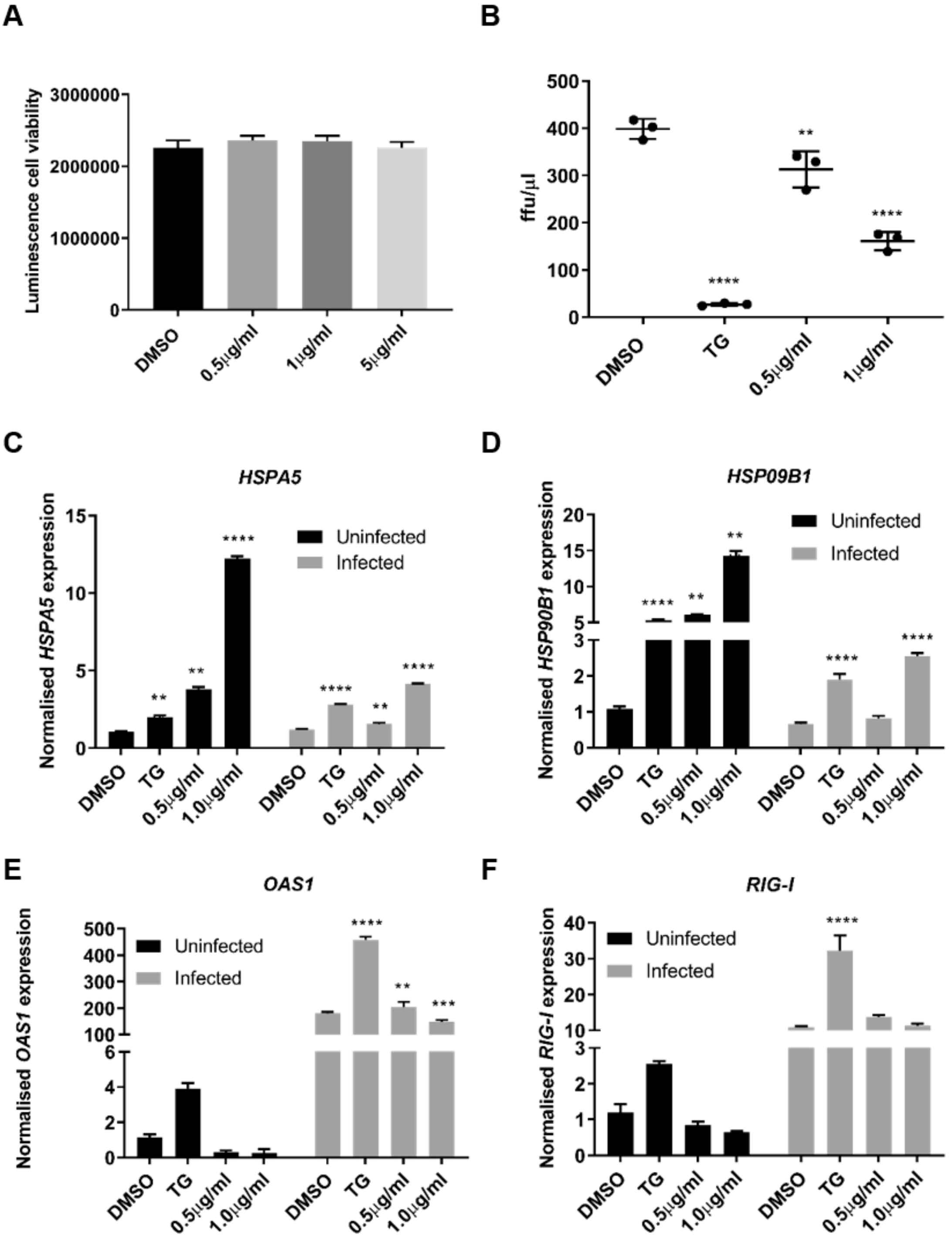

Figure S1. TG and tunicamycin mediated ER stress. (**A**) NPTr cells were incubated in indicated concentration of tunicamycin or DMSO control for 30 min and cell viability assay (CellTiter-Glo luminescent cell viability assay) performed 24 h later. Significance determined by one-way ANOVA, relative to DMSO control. (**B**-**F**) NPTr cells were primed for 30 min with 0.5 μM TG, tunicamycin (0.5 or 1.0 μg/mL) or DMSO, and subsequently infected with USSR H1N1 at 0.5 MOI for 24 h. (**B**) Spun sns were used in 6 h FFAs on MDCK cells. Significance determined by one-way ANOVA relative to the DMSO control. (**C**-**E**) Total RNA was extracted from each sample

to detect expression of ER stress markers (*HSPA5* and *HSP90B1*) and type I IFN associated (*OAS1* and *RIG-I*) genes, normalised to *18S* rRNA. Significance determined by two-way ANOVA, relative to corresponding DMSO control. *p <0.05 **p <0.01 ***p < 0.001 ****p <0.0001).
